# Health app policy: international comparison of nine countries’ approaches

**DOI:** 10.1038/s41746-022-00573-1

**Published:** 2022-03-18

**Authors:** Anna Essén, Ariel D. Stern, Christoffer Bjerre Haase, Josip Car, Felix Greaves, Dragana Paparova, Steven Vandeput, Rik Wehrens, David W. Bates

**Affiliations:** 1 House of Innovation, Stockholm School of Economics, Stockholm, Sweden; 2 Harvard Business School, Harvard University, Boston, MA USA; 3 Health Innovation Hub, German Federal Ministry of Health, Berlin, Germany; 4 Hasso Plattner Institute, Digital Health Center, Postdam, Germany; 5 Department of Public Health, University of Copenhagen, Copenhagen, Denmark; 6 Centre for Research in Assessment and Digital Learning (CRADLE), Deakin University, Melbourne, Australia; 7 Centre for Population Health Sciences, Lee Kong Chian School of Medicine, Nanyang Technological University, Singapore, Singapore; 8 Department of Primary Care and Public Health, Imperial College London, London, UK; 9 National Institute for Health and Care Excellence, London, UK; 10 Department of Information Systems, Centre for e-Health and Centre for Digital Transformation, University of Agder, Kristiansand, Norway; 11 beMedTech VZW, Strombeek-Bever, Belgium; 12 School of Health Policy and Management, Erasmus University, Rotterdam, The Netherlands; 13 Harvard Medical School and Harvard T. H. Chan School of Public Health, Harvard University, Boston, MA USA

**Keywords:** Health policy, Health services

## Abstract

An abundant and growing supply of digital health applications (apps) exists in the commercial tech-sector, which can be bewildering for clinicians, patients, and payers. A growing challenge for the health care system is therefore to facilitate the identification of safe and effective apps for health care practitioners and patients to generate the most health benefit as well as guide payer coverage decisions. Nearly all developed countries are attempting to define policy frameworks to improve decision-making, patient care, and health outcomes in this context. This study compares the national policy approaches currently in development/use for health apps in nine countries. We used secondary data, combined with a detailed review of policy and regulatory documents, and interviews with key individuals and experts in the field of digital health policy to collect data about implemented and planned policies and initiatives. We found that most approaches aim for centralized pipelines for health app approvals, although some countries are adding decentralized elements. While the countries studied are taking diverse paths, there is nevertheless broad, international convergence in terms of requirements in the areas of transparency, health content, interoperability, and privacy and security. The sheer number of apps on the market in most countries represents a challenge for clinicians and patients. Our analyses of the relevant policies identified challenges in areas such as reimbursement, safety, and privacy and suggest that more regulatory work is needed in the areas of operationalization, implementation and international transferability of approvals. Cross-national efforts are needed around regulation and for countries to realize the benefits of these technologies.

## Introduction

In various areas of health care, digital applications hold great promise for expanding access to services, substituting for and/or complementing existing standards of care, and creating value and convenience for patients. Yet globally, health care systems are struggling with how to incorporate and leverage the accelerating pace of innovation and commercialization of new digital tools that could potentially improve the treatment of illness and maintain health. Mobile health apps (health apps) epitomize this challenge.

While there is an abundant supply of health apps in the commercial tech-sector, the number of options represents a problem. Studies suggest that if confronted with too many choices, people struggle to make one^1^. A key challenge for the health care system is to facilitate the identification of safe and effective apps for health care practitioners and patients to generate the most health benefit as well as guide payer coverage decisions, where relevant^2–5^. Public debates across countries reveal a demand for ‘someone’ to provide a quality stamp on the apps that fulfil basic medical and privacy criteria, that is, to provide a labelling of apps that denote which have achieved standards or endorsement of some type^6^. The European Institute for Standardization has announced that it recommends that all countries should develop such a framework^7^. There is also a general trend toward patient empowerment, and this is especially apparent in this space. Some regulation is needed, especially for apps that involve the diagnosis, treatment, and/or management of chronic or high-risk conditions.

Nearly all governments are attempting to define policy frameworks that will be effective for improving health in this context. While such frameworks will not replace market-based evaluation mechanisms (e.g., stars in app stores), they can complement them and play an important role in providing guidance in this context. Our goal in this study was to compare the national policy approaches currently in development and/or use for health apps in nine countries with well-developed health care and regulatory systems, many of which are taking different paths. We also contribute forward-looking recommendations that may be helpful for guiding future policy developments in this area.

## Results

### Commonalities and differences in national approaches to health app policy

We identified a variety of approaches to the policy of health apps with some commonalities across the countries studied. Table 1 outlines the status of framework development in each country. As outlined in Table 1, Belgium and Germany have overall come the furthest in operationalizing and implementing their market access and reimbursement approval framework for medical apps (excluding apps that do not fulfil criteria for medical devices). In Germany, qualifying apps (known by their German acronym ‘DiGA’) are currently being evaluated through the ‘Fast-Track’ process, and those approved for use and reimbursement are made available in a central directory of digital health applications. Further, Belgium has implemented its mHealthBelgium validation pyramid, with 3 levels, each consisting of criteria related to regulatory issues (level 1), safe communication and privacy (level 2), and to financing and reimbursement (level 3). England’s emerging national approach, which includes assessment frameworks for evidence of effectiveness together with wider technical standards, also serves as a potential role model for many countries, although it, too, is under development, and does not yet incorporate reimbursement approval. In all other countries, initiatives to replace multiple local and fragmented initiatives with little impact with national frameworks are ongoing.

**Table 1:** National approaches to health apps policy (policy process).

Country	Actors developing national framework	Intended use of framework	Key regulations underpinning framework	National framework for market access approval developed	Reimbursement approval framework developed	End-user interface to clinical practice/ patients
Belgium (BE)	Initiative of the Federal Belgian Government. Three national authorities involved in defining the criteria are: Federal Agency for Medicine and Health Products (FAMHP), The eHealth Platform, NIHDI (National Institute for Health and Disability Insurance)	Centralized procedure run by 2 industry federations, namely beMedTech (medical technologies) and Agoria (representing companies in the technology sector)	EU Medical Device Regulation, Belgian law on data privacy	Yes, mHealthBelgium portal as quality seal listing all apps that fulfil criteria, on different levels (M1-M2-M3)	Yes, mHealthBelgium framework is unique path to submit financing/reimbursement dossier and all nationally financed/reimbursed apps will be listed there in level 3	Currently not applicable. The mHealthBelgium portal only lists all apps but prescription or activation is not happening via the portal
Denmark (DK)	Danish Medicine Agency (https://laegemiddelstyrelsen.dk/en/devices/new-tech-new-technological-possibilities-and-medical-devices (2020, as well as a collaboration of the Danish Health Data Authority, the Government, the interest organization for the five regions in Denmark, Danish Regions, and the interest organization of all the Danish municipalities, Local Government Denmark (Digital Health Strategy https://sundhedsdatastyrelsen.dk/-/media/sds/filer/english/digital_health_solutions/digital_health_strategy_2018_2022.pdf?la=da (2018))	Expected centralized approach	Danish law (Retsinformation https://www.retsinformation.dk/eli/lta/2008/1263 (2008)), EU regulations (Eur-Lex https://eur-lex.europa.eu/legal-content/EN/TXT/?uri=CELEX:32017R0746 (2017))	Not established yet, work in progress	Not established yet	Not established yet. Planned to be on the Danish e-Health Portal (https://sundhedsdatastyrelsen.dk/-/media/sds/filer/strategi-og-projekter/strategi-digital-sundhed/analyse-af-guide-til-sundhedsapps-04,-d-,01,-d-,19.pdf?la=da (2019))
England (EN)	Medicines and Healthcare Regulatory Agency, National Institute of Health and Care Excellence, NHSX	Centralized approach. Formal regulation of apps is done by the MHRA (medical device regulator). Adoption of technology is supported by NHSX – a national digital transformation agency for health. Health technology evaluation of apps –is done by NICE. Work is underway to bring these various approaches together as a simple and rapid regulation and access pathway.	UK Medicines and Medical Devices Act 2021 and UKCA marking	Yes, the Digital Technology Assessment Criteria (DTAChttps://www.nhsx.nhs.uk/key-tools-and-info/digital-technology-assessment-criteria-dtac/ (n.d.)). Standards for evidence of effectiveness and cost effectiveness of digital health technologies (provided by NICE https://www.nice.org.uk/about/what-we-do/our-programmes/evidence-standards-framework-for-digital-health-technologies (2021)). A joined up regulatory pathway and reimbursement framework are under development.	Not yet established – in development	https://www.nhs.uk/apps-library/
Germany (G)	Federal Ministry of Health (BMG) in collaboration with the Federal Institute for Drugs and Medical Devices (BfArM). Advising by the Health Innovation Hub (an independent think tank of the BMG, that advised the ministry on digital topics in particular from 2019–2021)	Centralized procedure run by the BfArM	German law (Digital Healthcare Act (DVG) and Digital Health Applications Ordinance (DiGAV)), EU regulations	Yes, implemented in the Fast-Track process run by the BfArM (https://www.bfarm.de/SharedDocs/Downloads/EN/MedicalDevices/DiGA_Guide.pdf (2021)); Apps are approved (provisionally or finally/permanently) for clinician prescription and patient use.	Yes, implemented in the Fast-Track process. Approval by the BfArM for listing in the DiGA Directory implies reimbursement approval for all apps that are prescribed to patients in the statutory health insurance system. Manufacturers’ list prices are paid for the first year of marketing after which time reimbursement amounts are governed by price negotiations between manufacturers and the National umbrella organization of statutory health insurers. (This approach mirrors drug reimbursement in the German system.)	Apps available by prescription (and thus reimbursed by the statutory health insurance system) are listed in the DiGA Directory (BfArM https://www.bfarm.de/SharedDocs/Downloads/EN/MedicalDevices/DiGA_Guide.pdf (2021)). Users can download these apps from traditional sources such as the App and Play stores, but require an activation code for use.
Netherlands (NL)	Ministry of Public Health, Well-being and Sports (VWS), Inspectorate for Health care and Youth (IGJ), The National Institute for Public Health and the Environment (RIVM), The Netherlands Standard Institute (NEN), National eHealth Living Lab (NeLL)	Expected centralized and federated approach.	Dutch law (https://wetten.overheid.nl/BWBR0023864/2019-07-01; https://wetten.overheid.nl/BWBR0040940/2018-05-25), EU regulations	Not established yet. Multiple guides for evaluating health apps developed, (e.g., Medical App Checker by Dutch Medical Association, and evaluation guide by the Dutch Community Health Services. These initiatives may be replaced by a Dutch version of the forthcoming CEN-ISO/TS 82304-2 standard	Not established yet	Not yet established. The Dutch Community Health Services runs an Appstore offering guidance regarding the quality of health app
Norway (NO)	Directorate of health, Directorate of eHealth and Norwegian Health Network (NHN) as central actors. Additional actors involved: Norwegian Smart Care Cluster and DNV-G (https://www.helsedirektoratet.no/tema/velferdsteknologi/rapporter-og-utredninger/Tryggere%20helseapper.pdf/_/attachment/inline/e3f6f78d-e56c-4c75-ba64-7bb37be4442c:a350b117c4f5ad0db055588fd58b01615e08c9c4/Tryggere%20helseapper.pdf).	In the short run, centralized, built upon the national citizen portal helsenorge.no and its existing tool catalogue. First step is to publish self-care and self-help health apps in an app library. In the long run, apps as medical devices to be integrated in public health services, also available on prescription as part of a central register on helsenorge.no.	Norwegian regulation (Medical Devices Regulation (MDR), In Vitro Diagnostic Regulation (IVDR), CE marking: General Data Protection Rights, ISO 27001 standard, Norwegian Code of Conduct), EU regulations.	Yes, work in progress as part of the “Safer use of health apps” national framework project. Approved apps will be published in an app library, building upon the existing tool catalogue at helsenorge.no. Apps will be validated with criteria relating to: usability and accessibility, data security, privacy and health benefits.	Not established yet	Limited number of apps available for prescription through helsenorge.no
Singapore (SI)	The Ministry of Health Singapore/Health Sciences Authority develops legislations concerning health apps as medical devices. Other stakeholders including industry associations, companies in the medical device industry and members of the public have been consulted (HSA https://www.hsa.gov.sg/docs/default-source/default-document-library/summaryoffeedbackreceivedfromthepublicconsulationontheproposedhealthproductsmedicaldevicesregulations_2aug07.pdf (n.d.))	Centralized. Apps must be approved by the HSA prior to releasing the app for use in Singapore. Approval process can range from straight-to-market, expedited review to full review (HSA https://www.hsa.gov.sg/docs/default-source/announcements-csg/press-releases/pr-final_mdlegislationchanges_22may2018.pdf)	Health Products (Medical Devices) Regulations 2010, Personal Data Protection Act 2012 (PDPA), Regulatory Guidelines for Software Medical Devices – A Life Cycle Approach and the National Telemedicine Guidelines (NTG) (non-legally binding)	Only for apps classified as medical devices. Depending on intended usage, such health apps need to meet standards outlined in the Regulatory Guidelines for Software Medical Devices: A Life Cycle Approach to be approved by HSA for use in Singapore. Alternatively, approval for use can also be fast tracked given a minimum of one out of five reference agencies’ approval (US FDA, European Notified Bodies, the Australian Therapeutic Goods Administration, the Japanese Ministry of Health, Labour and Welfare, and Health Canada), no prior rejection or withdrawal by/from any reference agency or HSA, no safety issues associated with the device globally and three years of marketing history (HSA https://www.hsa.gov.sg/docs/default-source/hprg-mdb/regulatory-guidelines-for-telehealth-products-rev-2-1.pdf (2019); https://www.hsa.gov.sg/docs/default-source/hprg-mdb/gudiance-documents-for-medical-devices/regulatory-guidelines-for-software-medical-devices---a-life-cycle-approach.pdf (2020))	Not established yet	There is no patient-facing portal compiling all approved apps. A database of registration information is available for public inquiry on the HSA and HealthHub websites (www.healthhub.sg). Health portals associated with the regulatory bodies e.g., HealthHub (https://www.healthhub.sg/Pages/apps.aspx) and Integrated Health Information Systems website (https://www.ihis.com.sg/Project_Showcase/Mobile_Applications (n.d.)) only feature health apps developed by public institutions under Ministry of Health’s supervision. Apps featured are recommended as helpful resources rather than a prescriptive element. There is currently no known effort to curate third-party apps to be recommended to healthcare providers and patients
Sweden (SE)	Swedish Institute for Standardization, Medical Products Agency, Health and Social Care Inspectorate, National Board of Health and Welfare, Data Protection Authority, Consumer Agency. The Swedish version of the ISO standard covers both wellness and medical device apps	No path chosen, and there is no obvious actor to use the criteria complementing MDR regulations. Semi-centralized approach where the Swedish Accreditation Agency will certify third party actors who can then approve wellness apps according to criteria discussed	Swedish legislation (Act (1993: 584) on medical devices. 1. Sweden (https://www.riksdagen.se/sv/dokument-lagar/dokument/svensk-forfattningssamling/lag-1993584-om-medicintekniska-produkter_sfs-1993-584); Law with supplementary provisions to the EU regulation on medical devices (SFS 2021: 600) (in Swedish) (https://svenskforfattningssamling.se/sites/default/files/sfs/2021-06/SFS2021-600.pdf?utm_campaign=lv_nyhetsbrev&utm_medium=email&utm_source=newsletter), EU regulations)	Not established yet. Extensive list of national criteria for evaluating quality of both wellness and medical device apps available, based on CEN-ISO/TS 82304-2 standard	Not established yet	Not established yet. One discussed idea is to add quality stamps (symbols) to apps, which may be available via different channels, e.g., Appstores and national health portal (1177.se), according to the emerging ISO standard
USA (US)	Food and Drug Administration (FDA)	Federated. Approval by the FDA for apps embedded in devices or which make normative recommendations (https://www.fda.gov/medical-devices/digital-health-center-excellence/digital-health-software-precertification-pre-cert-program (2021)). The FDA has taken a risk-based approach, and apps that are classified as medical devices and are of moderate or higher risk are subject to FDA regulation. Many wellness apps are not considered to be medical devices.	Local regulations	No national framework yet. A ‘fast-track’ pathway is in testing	Not established yet	Not established

Typically, several regulatory bodies are engaged, although one has primary authority, often with others covering specific aspects. For instance, healthcare supervision agencies, agencies responsible for market access and reimbursement approval of new medical procedures and products, and standardization bodies are often coordinating the initiatives in collaboration with actors representing patients (e.g., patient associations), professionals (medical professional societies), IT-vendor perspectives (industry representatives), actors responsible for data (e.g., in Sweden: data inspectorate), and consumer product approval (e.g., in the US: the Food and Drug Administration (FDA); in Sweden: Consumer Agency). The involvement of actors representing both clinical practice and consumer products is important in those countries where the frameworks are intended to cover both wellness apps and apps classified as medical products (SE, NL).

Pre-existing regulations (hard law in terms of legally binding legislation) in different domains influence frameworks (which represent soft law) being developed. Emerging frameworks are aligned with but serve to complement national legislation (e.g., regarding medical devices (see Supplementary Note 1), medical documentation, patients’/consumer rights, and data protection), as extant legislation has not typically been adjusted to the health app context, and additional guidance, policies, and clarifying regulations are needed. International regulations and standards primarily influence emerging frameworks in European countries. The emerging international ISO standard (https://www.iso.org/standard/78182.html), the GDPR and other EU regulations, such as the MDR, which came into full force in May of 2021 impact certain types of apps in European countries, while policies and guidance from the FDA and PDPA influence the US and Singapore, respectively (Republic of Singapore Personal Data Protection Act of 2012 https://sso.agc.gov.sg/Act/PDPA2012).

Most countries envision a centralized process in which one actor/committee will use the framework to evaluate apps (such as the ‘Fast-Track’ process in Germany, which is run by the Federal Institute for Drugs and Medical Devices (BfArM) and the same is applicable for the mHealthBelgium pyramid coordinated by industry federations beMedTech and Agoria). More decentralized approaches, for instance, where one accreditation agency will ‘certify’ those actors who can evaluate apps (e.g., the national accreditation agency (SE), or where local/regional/specialized actors will use available frameworks for their domain (NL) are also discussed.

Most countries envision approved apps that will be available through different channels, e.g., national health portals where they exist (e.g., DK, SE, NO), websites providing ‘catalogues’ or ‘directories’ of approved apps for specific domains (e.g., G, SI, NL), and commercial App stores—namely the App and Play stores (US), or a combination of these sources (e.g., in G and B where approved apps are centrally listed in the national portal but where access is given through the App and Play stores, though often only after a patient receives an activation code).

Supplementary Table 4 summarizes the content of existing/emerging frameworks in relation to five criteria for evaluating health apps suggested by Levine et al.^8^.

Existing/emerging frameworks include *transparency* criteria in terms of requiring information be made available to end-users about the intended use and purpose of the app, medical trials used to evaluate the app, compliance with GDPR (European countries), and/or national data legislation (e.g., G, B, EN). Some countries also ask for information about the manufacturer and its value proposition (e.g., EN). The emerging ISO standard (used as inspiration in e.g., SE, NL), suggests users should consent to advertisements and use of data and requires the description of the app (in e.g., an App store) to be ‘accurate and clear’ (https://www.iso.org/standard/78182.html (2021)).

Regarding *health content*, evidence supporting the intended use is required (sometimes both trials and ‘evaluations’ are allowed, and in some countries, several kinds of ‘positive effects’ (medical, structural, and procedural effects (Germany)), are allowed. EN requires different levels of evidence depending on the app’s purpose. The emerging ISO standard refers to the use of ‘appropriate’ peer-reviewed scientific literature in the development of the health app.

Regarding *technology*, emerging criteria include robustness and interoperability with EHRs (EN, SE, ISO). Some countries have used self-evaluation (i.e., app producers self-rate their qualities) for this but intend to move towards external evaluation (NL). The emerging ISO standard includes the criteria of application size (SE).

*Security/Privacy* is operationalized as compliance with national and EU legislation governing privacy and data-security (GDPR) (DK, G, EN, SE), and may be further specified at the national level (e.g., as in Belgium’s level 2). Further, Germany has implemented data protection laws that encompass, but go above and beyond the requirements of the GDPR. In the US, the Health Insurance Portability and Accountability Act (HIPAA) governs many forms of patient data, but is far less comprehensive than the GDPR, which takes a far broader definition of ‘personal data’ and ‘data concerning health’. Some countries are discussing requiring the use of industry standards in risk models for vulnerability testing and the implementation of ISO/IEC 27001 or recognized equivalents by the health app manufacturer and all organizations providing associated services (NO). The emerging ISO standard suggests criteria such as protection against theft and viruses, signalling of breaches, authentication, data sharing, and maintenance (ISO, SE).

*Usability* is considered in EN in terms of demonstration of user-centred design, accessibility standards (WCAG), and development with iterative/agile principles. Proof of ‘ease of use’, defined as ‘intuitive usability and learnability of the [app] for the target groups addressed’ is a requirement for all apps in the German DiGA directory. The emerging ISO standard (SE, NL) considers functionality, aesthetics, and availability in multiple languages.

## Discussion

We compared health policies regarding health apps across nine countries. Our results demonstrate that countries are at different stages of development and vary in the degree to which the evaluation of health apps is envisioned to be centralized, but there are several commonalities, including ongoing initiatives involving a set of national agencies, and the use of relevant existing and emerging international regulations (Table 1). There is great interest in the use of apps in all the countries evaluated, but even Belgium, Germany and the UK, which are relatively far along in their operationalization of frameworks, are struggling with efficient implementation. We acknowledge, though, that this is a space in which the power of individual governments may be limited and there is ongoing disintermediation of traditional gatekeepers such as healthcare organizations. Below, we discuss implications of the current developments and focus on points around which we believe international collaboration might be beneficial. We focus our discussion on two major types of future challenges: first, challenges tied to the implementation of frameworks; and second, challenges tied to specific criteria dimensions.

While previous work has highlighted the *need* for frameworks^2–5^ or focuses on evaluations in single countries^6,9–11^, we focus on showing the actual status of digital health app regulation in nine countries and three regions of the world. There are many reasons that international collaboration among researchers and policy makers could be valuable in this area, with an obvious one being that health apps do not respect national boundaries. The potential users of English-language apps, for instance, are not only consumers/patients in nations where English is the primary language but also individuals anywhere in the world who have English language proficiency. Thus, the expanding use of health apps within and beyond formal healthcare—and the information health exchanges this involves—implies a diminishing of the power of traditional (national) gatekeepers of medical information (and national governments). Standards and soft policy can play an important role here–but we expect the disintermediating to continue irrespective of standards. Monitoring, adjusting, experimenting with how to address this to ensure safe and effective health apps is thus a key challenge.

As regards challenges related to the implementation of the emerging frameworks, most countries have opted for centralized approaches to evaluation. This is preferable to self-evaluation. However, centralized approaches also run the risk of creating bottlenecks, a risk that seems important given the vast and growing supply of health apps but a low number of apps that are currently ‘approved’ in countries having a well-developed process (for example, only 20 apps and 1 app had been approved for reimbursement in G and B respectively, as of the beginning of September 2021). For this reason, a more decentralized approach, such as the ‘accreditation’ of evaluation agencies may be a viable solution (as discussed in Sweden).

Countries that are further along in their operationalization limit their approvals to health apps meeting criteria for being defined as medical devices. This necessarily excludes certain products that are not classified by the International Devices Regulators Forums as ‘Software as a Medical Device’ (non-SaMD) products and raises the question of how wellness apps—that do not fulfil these criteria but can still create demonstrable value for patients—should be vetted (see Definitions below and Supplementary Note 1 for additional detail). The emerging ISO standard, which provides visual symbols representing different aspects and degrees of quality to be displayed within apps (thus providing guidance to users independently of through what store/website the app is found), and other creative approaches, such as ‘nutrition labels’ for direct-to-consumer apps^12^, much like other food product regulation which have been proposed by digital health researchers but have not yet been implemented, can provide valuable guidance here.

In most countries, a combination of approaches will most likely emerge, wherein some apps with intended use in clinical/self-care practice will be formally evaluated and displayed as formally ‘approved’ or ‘authorized’, while other wellness-oriented apps may be subject to market-based evaluation by potential consumers. Yet a basic level of quality check is important even for wellness-oriented apps. The platforms that enable sales of these such as the established App and Play stores, therefore, hold an important role in conveying information about apps—if not formally ‘approving’ them—before displaying them in their stores. While these intermediaries have several criteria in place (Supplementary Table 5 outlines the criteria used by Apple’s App and Google’s Play store), research shows that an alarming number of low-quality apps pass through them^13–16^. Hence, having a third party play this role, using for instance the emerging ISO standard, or potentially through standardized labelling requirements or crowdsourcing, would be an alternative.

Overall, a middle road may make sense in many cases, for example when risk is low, the bar for approval is low—although, in Europe, the MDR makes it likely that most apps that meet the definition of SaMD will be (more stringently than previously was the case) classified as at least Class IIa medical devices and therefore subject to regulation^17^. For apps that help to manage or treat chronic conditions which are responsible for a large proportion of healthcare costs and require long-term use or involve high-severity short term conditions an additional certification of some sort, perhaps by a third party, is almost certainly advisable and regulatory approval should be required for those that are high risk (such as suicide prevention apps^13^). This approach is consistent with the ‘risk-based framework’ outlined in U.S. Food and Drug Administration (FDA)’s Precertification (‘Pre-Cert’) Program for Digital Health Products. Although Pre-Cert is still in its pilot phase, it is expected that it will expand to include other companies and software products beyond those meeting the definition of SaMD in the future (https://www.fda.gov/medical-devices/digital-health-center-excellence/digital-health-software-precertification-pre-cert-program (2021)).

More broadly, as countries are expected to use different combinations of evaluations by central ‘trusted’ actors and market-based approaches, there will be an opportunity for cross-country knowledge exchange among researchers and policy makers, focusing on how centralized and market-based approaches can co-exist and complement each other, and the throughput vs. trust in evaluations achieved in countries using different approaches.

Regarding the specific criteria for app evaluation in specific domains, several questions warrant further attention among actors developing, implementing and using such frameworks at the national and international levels. For instance, while most countries consider *‘transparency’*, which is a prerequisite for informed consent, this domain may need further specification. As noted in the recent piece by Grundy et al.^18^, apps currently provide alarmingly low levels of information to consumers about data use. Against this backdrop, ‘use of data’ (as suggested by the emerging ISO standard) is thus welcome but could mean many things. While most discussions concern the need for informing users about the immediate use of the data the apps collect and generate about them, allowing patients to consent to *reuse* by additional actors may further be equally important, as patients may want to make their data as impactful as possible while ensuring privacy. Transparency regarding ‘how the app achieves its decisions’ (suggested by ISO) is similarly critical but needs to be better operationalized in all countries, not least in relation to the increasing incorporation of AI-based algorithms in apps.

With respect to *health content*, approvals in pioneering countries such as Germany have been based mainly on medical trials. As many apps aim at creating structural and procedural improvements to care (e.g., patient literacy, improved interaction between physicians and patients, improved sense of control among patients in self-management of their disease, etc.), different types of evidence beyond RTCs may be required. Real-world data (RWD) and real-world evidence (RWE) are expected to play a role in the ongoing evaluation of apps in practice^5^. Further, whether medical evaluations done in one country will be considered valid evidence in others is a further reasonable and legitimate source of uncertainty. So far, national approval has been based on medical trials in a given country, but, for small countries or products with a small patient population (e.g., those targeting individuals who suffer from a rare disease) this may not be practical, and such a requirement also creates a significant burden for companies.

Another dimension of health content involves the degree to which information is presented in a clear and accurate way on the app to its different audiences (purchasers, patients, or health care professionals). This aspect, which refers to information quality more than the evidence supporting the clinical value of the app, is largely non-operationalized in existing/emerging frameworks, although Germany does require manufactures to present proof of the basic quality of medical content and usability, which combined can address this challenge for regulated apps (see Supplementary Table 4). This aspect is pertinent to address more in frameworks given that recent studies^19–21^ identify numerous safety concerns relating to the quality of information presented by apps.

A key issue regarding *technological interoperability* is whether data can be exchanged with electronic health records. This is considered in several existing/emerging frameworks (EN, SE, G, ISO). Yet, so far, most apps do not exchange data, even though this could potentially be highly beneficial, especially for chronic disease management. Most EHRs now do have open APIs, which should make exchange possible. In Germany, interoperability is a requirement of the Fast-Track and further compatibility with the newly introduced e-prescription system and the electronic patient record is also planned. B and EN also have this requirement, but all countries experience struggles with how to implement automated tests in this area.

*Security/privacy* in Europe is defined in terms of GDPR compliance, while in the U.S. HIPAA is the relevant statute. While most apps do ask patients whether they consent to secondary use of their data, often this consent is buried. Further, while criteria such as protection against theft and viruses, signalling of breaches, authentication, data sharing, and maintenance work are discussed, there is uncertainty regarding their operationalization. Indeed, among traditional (regulated) medical devices that contain software, it is already known that there is a deficit in the provision of information about cybersecurity in publicly available documents, suggesting that more regulatory guidance and/or public policy is likely needed in this area^22^.

*Usability* represents a critical concern, as many apps score poorly in this regard, and many are especially inaccessible to patients with low language skills or literacy, even though this group may particularly stand to benefit. It is also unclear what group(s) or institution(s) should evaluate app usability, as perceptions of this could differ markedly among patients with different conditions and experiences. This is perhaps where an ongoing crowdsources/market-based rating system could particularly complement initial evaluations performed by centralized actors, even though it would create a risk of gaming.

Little attention to date is given to criteria and standards for continuous *updating* of health apps, in terms of software performance, content, as e.g., new guidelines emerge and evidence for latest best evidence for mobile interventions, e.g., on human-computer interaction, AI or meaning of sensor data. This domain requires attention especially as this is an emerging and dynamic field where not only medical evidence progresses but even faster mobile phones, their operating systems, features and functionalities. This is critical also considering the evidence^19^ suggesting that apps on the market exhibit many flaws in software functionality, which could potentially be addressed in updates.

Finally, *accountability* (for not only medical effects but also for side effects of apps and changes in consumption patterns triggered by the app)^23^ and the related issue of the *integration of apps into care pathways*^24^ are two dimensions of critical importance for the effective implementation of apps. These issues are lacking in most emerging frameworks and warrant future attention. ISOs criteria for ‘social benefit’ (5.2.5) (https://www.iso.org/standard/78182.html (2021)) and B:s requirements for level 3 can here be used as inspiration, as it encourages app developers to include economic analyses that consider healthcare savings in other healthcare settings than where the cost is generated, and benefits for society more broadly.

Overall, a balance needs to be achieved between detailed evaluation criteria on the one hand, and the applicability of frameworks on the other. As most emerging/existing frameworks represent soft law (not legally binding but based on voluntary use) and/or early-stage programs or pilot projects, usability (also considering the time and other costs of using them) of the frameworks is important too. This again, speaks for a staged model with different ‘levels’ of approval, in order to strike a good balance between product risk and information/regulatory requirements.

In summary, while ongoing initiatives in many countries are ambitious and continue to make progress in the service of bringing better products to patients, the effectiveness of approaches in use is uncertain, as the operationalizations of criteria to date are rarely sufficiently specific to offer providers and patients the guidance they need to make evidence-based decisions about apps.

Current end-user interfaces are especially immature. Ideally, a clinician seeing a patient with a chronic or acute condition might have available a portfolio of favoured/vetted/approved apps that they could pick from, based on the patient’s characteristics. A clinician should also be able to prescribe an appropriate app for the patient, making it easy for the patient to access the intended product, which in most instances would interoperate with their electronic health record^24^. Indeed, this is the goal (and a key early success) of the German system for regulating and approving ‘prescribable apps’ for individuals in the statutory health insurance system.

Patients would like to be able to search for certified apps (fulfilling basic criteria) for both health and wellness as well as chronic conditions that they feel best meet their needs. For example, a patient with diabetes might opt to try several apps, ultimately settling on the one they find most motivating. Thus, the presentation of app quality to end-users is critical. If apps are to be provided through multiple channels, the apps themselves may need to include information about their quality level and other features such as data privacy and security. The emerging ISO standard provides a symbol system with visual ‘labels’ to be displayed in the app, to guide users. This may be viable—assuming it becomes a standard implemented globally. Search functions need to complement this, allowing users to search for apps for specific intended uses, with well-established minimal levels of quality.

Payers are another group facing the selection challenge. Like the above, guiding interfaces facilitating the identification of meaningful apps from the perspective of this groups are also needed. These actors act as gatekeepers since apps that do not pass their filters will not be widely used while those that do may have large audiences.

From the app manufacturer’s perspective, such criteria may or may not be welcome, and many developers may be concerned about barriers to entry that they perceive to be ‘too high’. But manufacturers would likely welcome approaches that enabled approvals in one country to be transferrable or expedited in others. Regarding transparency, privacy, and security, compliance with GDPR provides some degree of transferability across European countries. But in other contexts, criteria and associated regulations are sometimes primarily available in the native language (e.g., Germany). This is a key issue for app producers in small countries.

In this study, we have explored the current policies around the app marketplace across nine countries and based on this evaluation provide a set of recommendations and common issues for consideration. Health apps and the exchange of data between new actors will continue to undermine the power of traditional gatekeepers. Nonetheless, national standards could play an important role in creating awareness in markets, setting norms, and safeguarding basic quality dimensions. Clearly, this market is increasingly global and international collaboration could be beneficial in many ways, for example around issues of app transparency, health content, technology, and security/privacy. Further efforts of international researchers, practitioners, and users to identify and articulate common issues across countries as well as important settings for policy evaluation will therefore be vital to the ongoing growth and development of this nascent setting with great potential to improve care in new ways.

This study has limitations. This is an exceptionally broad area of health technology and we elected to focus on apps rather than digital health more broadly. We included only a small number of countries, but we intentionally selected a diverse group of settings that are some of the most advanced with respect to the regulation and use of health apps.

## Methods

We evaluated seven European countries, as well as the United States and Singapore. We purposively selected countries that have made at least some progress in this area, but with varying approaches. For pragmatic reasons, we excluded countries to where we had no direct access. The following nine countries were included: Sweden, Norway, Denmark, Netherlands, Belgium, Germany, England, the United States, and Singapore. See an overview of participating countries in Supplementary Table 1. Drawing on previous studies involving cross-country comparisons of regulatory (policy) approaches to health IT^25^, we initially approached the nine different national policy contexts based on an analytic model for policy analysis^26^ that distinguishes between policy context, policy process, and policy content (see Supplementary Table 2). This study focused on policy content: who/what is regulated^26^. We limited our focus to national-level policy developed by the government, governmental agencies, and national standard-setting bodies. We excluded frameworks developed by regions or local, specialised communities.

### Definitions

#### Health app

We define a health app as a computer program or software application (designed to run on a mobile device) “intended to be used specifically for managing, maintaining, or improving the health of individual persons, or the delivery of care” (ISO https://www.iso.org/standard/78182.html (2021), p 5). Health apps may target specific medical conditions and clinical practice areas, or they may be generic, aiming to improve health and wellness more generally—for example, by facilitating communication between patients and clinicians, etc. There are professional as well as patient-facing apps. We incorporate both software (SW) and data generated/stored by the app in our definition of a health app. Hardware was excluded from consideration, although health apps can be used on various devices including smartphones and personal computers. A health app may be categorized as ‘Software as a Medical Device’ (SaMD) if it meets relevant criteria and may thus be subject to medical device regulations^17^, in particular the EU’s Medical Device Regulation (MDR, see Supplementary Note 1 for additional detail). The International Medical Devices Regulators Forum defines SaMD as ‘software intended to be used for one or more medical purposes that perform these purposes without being part of a hardware medical device’ (IMDRF http://www.imdrf.org/docs/imdrf/final/technical/imdrf-tech-131209-samd-key-definitions-140901.pdf (2013)). We focus here on the policies intended to complement extant international and national medical device regulations. We excluded frameworks developed by specialist/patient communities and frameworks developed at local and regional levels.

#### Approval—market access

the permission to introduce the app to the market. (For instance, through an app store, independently or whether it is used by a clinician or not—it could be used by a citizen for private purposes only). *Reimbursement approval*: the permission and/or mandate to reimburse for use of the app by (typically tax-funded) third party purchasing actors such as insurers/regions/states. Both market access and reimbursement approval are part of what we refer to as the *policy approach* to apps. We exclude the various efforts made by interest groups, industry stakeholders, and associations, focusing solely on governmental initiatives here.

#### Policy

We distinguish between ‘soft’ and ‘hard’ regulation. While there is no easy dividing line between soft versus hard regulation, we defined hard law as national legislation that is mandatory and absolute (also referred to as binding or rule-based governance), and soft law as alternative forms of governance, which are conditional or voluntary. Soft law refers to rules that are not legally binding, for example, recommendations, agreements, national action plans, or policy documents. Soft law entails normative commitment and may have political effects^27,28^. This implies that soft law shall be considered politically binding rather than legally binding. While soft law is sometimes referred to broadly as regulation that relies on open-ended processes such as benchmarking and peer group audit, we have only included national recommendations here.

### Data collection

Document review and analysis constituted the primary source of data in the study. Based on the guiding definitions above, participating researchers gathered documents from their respective countries describing hard and soft laws relating to each country’s policy approach to health apps, as of mid-2021 (Q2–Q3). Dr. Stern who is currently based in Germany collected data about it given her expertise in their Fast-Track process. Documents included healthcare legislation, national strategies and e-Health reports, technology and e-service standards. We also performed interviews with key individuals involved in developing frameworks (*N* = 14) (See Supplementary Note 3). Selected parts of the vast amounts of information gathered about each country were translated by the authors from the local language into English and sorted in terms of a framework with separate categories for policy context, process and content, and sub-categories representing the target of the regulation (possible criteria dimensions). Each author completed the framework for their respective country, which was double-checked and revised by the first and last author at each stage of data collection and revision. Work on the table content was iteratively completed by all authors and updates were communicated through September of 2021. Subsequent changes in regulations were not included.

### Reporting summary

Further information on research design is available in the Nature Research Reporting Summary linked to this article.

## Supplementary information

Supplementary Information

Reporting summary

## Data Availability

The datasets generated during and/or analysed during the current study are available from the corresponding author on reasonable request.
