# Supplementary material for: Health app policy: international comparison of nine countries’ approaches
Source: NPJ Digit Med. 2022 Mar 18;5:31. doi: 10.1038/s41746-022-00573-1 (PMC8933556; doi:10.1038/s41746-022-00573-1)
Supplement: Supplementary file 1 — Supplementary Information [file 41746_2022_573_MOESM1_ESM.docx]

**SUPPLEMENTARY INFORMATION**

**Supplementary Table 1. Overview of participating countries (policy context).**

| **Country** | **No. of citizens (million)** | **Organization and funding of healthcare** |
| --- | --- | --- |
| Belgium | 11.0 | Tax-funded mandatory insurance with choice among nonprofit insurers (‘sickness funds’). Individuals can opt into additional coverage by the private health insurance system, |
| Denmark | 5.6 | Tax-funded. Regional and municipal governments are responsible for provision of care and may contract public and private providers. |
| England | 55.0 | Centralized single payer health system – National Health Service – funded from general taxation. Free care for acute medical care. System provided by local configurations of purchasers (clinical commissioning groups) and providers (trusts and primary care), with some centralized purchasing for rare/high-cost conditions. Currently undergoing restructuring to increase integration between providers and purchasers. |
| Germany | 83.0 | Mandatory insurance with patient choice among nonprofit insurers (‘sickness funds’). High-income individuals and public servants can opt into the private health insurance system, which covers roughly 10% of individuals. |
| Netherlands | 17.0 | Mandatory private insurance. Private care providers deliver care. Supervision of health system is conducted through independent bodies. |
| Norway | 5.4 | Tax-funded. Regional and municipal governments are responsible for provision of care and may contract public and private providers. |
| Singapore | 5.7 | Mandatory basic (tax-funded) public healthcare insurance for all citizens and permanent residents (MediShield Life) and a compulsory nation-wide tiered savings scheme for employed Singaporeans/permanent residents. Optional private insurance plans targeted at extended coverage to private hospitals and clinics also exist. |
| Sweden | 10.0 | Largely tax funded. Regional and municipal governments are responsible for provision of care and may contract public and private providers. |
| United States | 320.0 | Public and private insurance plans contract private physicians and hospitals. Insured patients pay noncovered costs out-of-pocket; 8–9% of the population uninsured. |

**Supplementary Table 2. Data collection framework: templates used.**

***Policy context, process, and content (Walt & Gilson 1994).***

| **POLICY CONTEXT AND PROCESS** | |
| --- | --- |
| **Actors involved in developing framework** | What authorities/stakeholders are the governmental actors involving when developing frameworks (patient associations, tech-firm alliances etc.)?  At national/regional-level (or international) level?  Are the actors departing from an existing framework? |
| **Intended use/operation of framework** | Is centralized use of the framework expected (‘national’ funnel), or decentralized (clinical/individual level/crowdbased/self-declaration/combination) |
| **Key regulations influencing/underpinning frameworks (hard law)** | National/international regulations (e.g., GDPR)  Existing/expected forthcoming regulations |
| **POLICY CONTENT** | |
| **Use approval framework developed for clinical practice (physician can use app together with/inform/or prescribe app)** | On basis of what criteria are health apps permitted to be introduced on the market (e.g., via an app store)?  Are further criteria being developed to help patients/clinicians choose among the various apps that pass the minimal requirements allowing them to be available in ‘app stores’? This could be, for instance, related to efforts to display certain apps on national health portals. If so, what criteria are being developed – focusing on what? See list of potential areas in Table 3 below.  Stages in approval process?  How often are ‘candidates’ vetted? Is ongoing evaluation/evidence required beyond the initial decision?  Enforcement: is there a process for withdrawing approval of apps/sanctions?  What, if any, technological solution underlying approval decisions is envisioned? For instance, are apps supposed to submit a request to platform that ‘vets’ app in an automated manner? |
| **Reimbursement approval framework developed** | Same as above |
| **Interface to clinical practice/patients** | Are ’approved’ apps presented on a specific portal/any portal/app store but with a ’visual stamp’?  If yes:  Are approved apps searchable/findable/possible to ’order’?  Are their prescription possible to integrate with EHRs?  Is there anyone responsible for educating users? |
| **Additional aspects** | Are countries focusing on certain type of apps? Targeting certain medical conditions? Are countries categorizing apps based on medical or other attributes? |

***Mobile health app rating domains and criteria, adapted from Levine et al.^13^.***

| **Domain** | **Criteria** |
| --- | --- |
| Transparency | Cost of app (purchase price, subscriptions, in-app purchases)   - Are the prices, subscriptions, and in-app purchases accurately conveyed?   Consent   - What is the quality of the consent process, if any? - Financial disclosure (no black-boxed selling of users’ personal data to fund app)   Accuracy of app store description   - How accurate is the app store description of the app’s purpose? |
| Health content | Is the app backed up by evidence?   - e.g., trials   Appropriate measurement   - Does the app appropriately measure what is claims to measure?   Appropriate interpretation of data   - Does the app appropriately interpret what is claims to interpret?   Quality of information   - How high is the quality of information?   Potential for harm   - Is the potential for harm minimized?   Literacy level   - How appropriate is the literacy level for the app’s intended audience?   Presentation of information   - Is information presented in an optimal manner? For ex, is scaffolding used? |
| Technical content | SW performance/stability   - Does the app run well with zero interface crashes or bugs?   Interoperability   - Is the app able to exchange information with EHRs and other apps?   Bandwidth   - Does the app require significant bandwidth to run?   Application size   - Does the app require significant storage capacity? |
| Security/Privacy | Does the app comply with data protection laws?  Protection against theft and viruses   - Does the app follow best practices in security with optimal anti-virus and safeguards against breaches, hackers, and damage?   Signalling of breaches   - If a breach occurs, does the app have a method to notify its users?   Authentication   - Is the authentication procedure appropriate?   Data sharing   - Does the app support data reuse (e.g., by making data findable and using standard tagging), and when sharing information, does the app use best practices? (e.g., ask users for permission).   Maintenance   - Does the app have regular cycles to update and patch its security?   Anonymization   - Does the app appropriately anonymize individuals? |
| Usability | Installation and setup   - How easy is installation and setup?   Functionality   - Quality of ease of use, navigation, gestural design, help/instructions?   Aesthetics   - Quality of layout, graphics, visual appeal, and image readability?   Customization/tailoring   - Ability to customize and tailor to the specific user's needs?   Ease of use for users with low literacy and numeracy  Available in multiple languages |

**Supplementary Table 3. Table with people interviewed (*N*=14)**

All interviews were performed during 2021.

| Country | Position of person interviewed | Approximate length of interview/email correspondence |
| --- | --- | --- |
| Sweden | Quality coordinator at Swedish Medical Products Agency | 30 min |
|  | Project leader of Swedish ISO committee on health apps | 60 min |
|  | Assessor within the Department of Medical Devices | Email correspondence |
|  | Project Manager in Healthcare | Email correspondence |
| Denmark | Consultant in Mental Health or Psychiatric Services in the Region of Southern Denmark | Email correspondence |
|  | Department leader of The Danish Health Data Authority | Email correspondence |
|  | Administrative officer at the Danish Medicine Agency | Email correspondence |
|  | Business consultant at Sundhed.dk | Email correspondence |
| Netherlands | ISO Expert Health App Assessment; National eHealth Living Lab | 60m |
|  | Medical researcher, National eHealth Living Lab | 60m* |
|  | Associate Professor E-Health and Regulation | Email correspondence |
|  | Medical researcher working with health apps in practice | 60m |
| Norway | Product responsible for the tool catalogue at helsenorge.no | 60 min |
|  | Legal consultant at helsenorge.no | 60 min |

**Supplementary Note 1. The European Union’s Medical Device Regulation.**

Regulation (EU) 2017/745 of the European Parliament and of the Council, known as the European Medical Device Regulation (MDR) defines a medical device as:

Any instrument, apparatus, appliance, software, implant, reagent, material, or other article intended by the manufacturer to be used, alone or in combination, for human beings for one or more of the following specific medical purposes: Diagnosis, prevention, monitoring, prediction, prognosis, treatment, or alleviation of disease … and which does not achieve its principal intended action by pharmacological, immunological, or metabolic means, in or on the human body, but which may be assisted in its function by such means … (MDR, Article 2(1) https://eur-lex.europa.eu/legal-content/EN/TXT/PDF/?uri=CELEX:32017R0745)

In EU member states, all products that manufactures intend to place on the market and meet the EU’s definition of a medical device are required to comply with the MDR, which came into force in May of 2021.

Brönneke et al.10 compare the MDR’s definition of a medical device to that of the U.S. FDA and discuss its implications for manufacturers – including the process of conformity assessments via EU Notified Bodies – in detail.

Importantly, the MDR increases the requirements for software products – specifically *those apps that meet the definition of a medical device –* to fulfil the conformity assessment required for a so-called CE (*conformité européenne*) Mark.

For additional details on the MDR, the authors of this article recommend the resources and background information provided by German’s Johner Institute at: <https://www.johner-institute.com/articles/regulatory-affairs/medical-device-regulation/>

Notably, there are numerous software apps that are used by professionals that are SaMD but not consumer facing.

**Supplementary Table 4. Health app quality criteria dimensions (adapted from Levine et al. 2020).**

| Country/ Criteria | Transparency | Health content | Technical content | Security/Privacy | Usability |
| --- | --- | --- | --- | --- | --- |
| Definition of criterion | Info about app to end-user, basis for consent | Evidence basis, appropriateness of measurement and interpretation | Software performance interoperability/ bandwidth requirements/ application size | Authentication/ anonymization, Protection against theft, viruses/signalling of breaches/data sharing/maintenance | Ease of installation and use/ tailoring/multiple languages |
| Belgium | Each app listed in the mHealthBElgium portal reveals information about target audience, prices, user instructions, connectivity to sensors and how they comply to the dedicated criteria in each level. | Cfr Germany | To reach level 2 within mHealthBelgium pyramid, different ICT criteria need to be met, e.g., regarding technical and semantic interoperability. | To reach level 2 within mHealthBelgium pyramid, different ICT criteria need to be met, e.g., regarding data privacy, authentication, identification as well as therapeutic relationship and informed consent | No hard checks are done. The end user will in the end judge whether he want to use the app or not. |
| Denmark | Apps must comply with Qualification and classification of software - Regulation (EU) 2017/745, Regulation (EU) 2017/746, GDPR and Danish Law. Other criteria are unknown. | Unknown. | Unknown | Apps must comply with GDPR and Danish Law | Unknown |
| England | Digital Technology Assessment Criteria (DTAChttps://www.nhsx.nhs.uk/key-tools-and-info/digital-technology-assessment-criteria-dtac/ (2021)) include specific but nonassessed sections on information about the company providing the product and the value proposition. | Effectiveness and cost effectiveness of Apps are evaluated by NICE (where they have been selected for assessment). Evidence standards for digital health technologies are described in the NICE Evidence Standards Framework (<https://www.nice.org.uk/about/what-we-do/our-programmes/evidence-standards-framework-for-digital-health-technologies> (2021)), which requires evidence proportionate to the function of the app. Safety is regulated by MHRA. | Evaluated with the DTAC standard – which includes 1) interoperability questions about capability to read/write into EHRs using industry standards, 2) standards on APIs and compliance with specific ISO/IEEE standards where appropriate. DTAC also contains specific reliability standards. | Apps must comply with current English legal basis for data protection.  DTAC standards include 1) provision of a data protection impact assessment 2) self-assessment against common cyber threats 3) should penetration testing (OWASP) and 4) load testing – amongst other technical standards | Evaluated against the DTAC standard which includes specific standards on usability and accessibility, including demonstration of user centred design, accessibility standards (WCAG), and development with iterative/agile principles. |
| Germany | List of the apps in DiGA registry include prices, information on the clinical trial(s) used to evaluate the app, platforms on which the apps are available, and special specifications that apply to the app. | The developer must show proof of quality of medical content, compliance with general requirements of safety and ‘positive care effects’ (medical benefit or structural and procedure effects^25^). The Fast-Track process facilitates broad flexibility in evidence generation: can use a variety of study designs, but must designs broadly constitute some form of quantitative, comparative study showing that the application of a certain digital health application (usually in addition to standard care) is better than the absence of its application. Explicitly accepts retrospective studies, such as cohort studies, as sufficient evidence, if meaningful data are used and if the study population is comparable with the actual intended users. | The developer must show proof that the app meets pre-specified standards with respect to functionality, robustness, and interoperability. | The developer must show proof that privacy and data-security regulations are met. A checklist for manufacturers is available prior to submission to the Fast-Track process. | The developer must show proof of user friendliness (to patients and, where relevant, clinicians). |
| Netherlands | Initiatives for uniform evaluation criteria developed by various professional associations in medicine, public health and mental healthcare (e.g., the App Checker). Different domains are developing their own overviews: medicine (KNMG), public health (Community Health Services) and mental health (MIND, the Dutch Platform for Mental Health). To be further developed when CEN-ISO / TS 82304-2 is published. | Self-evaluated through the App Checker or app evaluated by the Community Health Service. To be further developed according to the CEN-ISO / TS 82304-2. | Self-evaluated by app producer through the App Checker or app evaluated by the Community Health Service. To be further developed when CEN-ISO / TS 82304-2 is published | Self-evaluated through the App Checker or app evaluated by the Community Health Service. To be further developed when CEN-ISO / TS 82304-2 is published. | Self-evaluated through the App Checker or app evaluated by the Community Health Service. To be further developed when CEN-ISO / TS 82304-2 is published. |
| Norway | Not defined at the moment. For the current tool catalogue published at helsenorge.no, available information about the health apps are: short description, purpose of usage, keywords, vendor, and information about switching the citizen to the terms of use in that particular app. Consent is regulated separately depending on the app in question. In order to use these health apps, citizens have to accept the terms of use in the particular app and can withdraw their consent at any time. | Clinical content to be evaluated based on: involvement of doctors/medical personnel in the development of the app content, patient contribution in the development of the app, overseeing of the development by authorities/public institutions and available documentation in the form of qualitative studies that support the clinical effect of the app. | The design and development of the app should follow a recognised app design standard, such as WC3, WCAG 2.1 AA or AAA, ISO 9241, Apple HIG, or Android App Quality Guidelines. | Data security and privacy are treated as separate assessment criteria. Security criteria relate to: usage of industry-standard for the risk model in the PEN/Vulnerability testing; implementation of ISO/IEC 27001 or a recognized equivalent by the health app manufacturer and all organizations providing associated services; and Security Assesment.  Privacy criteria refer to: data processing overview explanation of which personal data is collected for which purpose and on which systems;  Privacy Policy which should be clearly available and regularly updated; named Data Protection Officer (DPO), responsible for the legal and regulatory compliance of processing personal information. | The app should respond to device preferences (such as change font/size), provide support for users with dissabilities such as: poor sight, or hearing difficulty. |
| Singapore | Apps qualified as medical device is to follow regulations as specified in Medical Device Guidance GN-08: Guidance on Medical Device Advertisements and Sales Promotion for app description on app stores (HSA . <https://www.hsa.gov.sg/docs/default-source/hprg-mdb/gn-08-r2-guidance-on-medical-device-advertisements-and-sales-promotion.pdf> (2018)). Beyond this, no current regulation concerns transparency of app pricing and financial disclosure. Consent to app use of any kind is governed by the Personal Data Protection Act 2012. | For apps qualifying as medical device, clinical quality and risk level associated with app use are to be evaluated following Heath Sciences Authority Singapore’s Regulatory Guidelines for Software Medical Devices: A Life Cycle Approach (HSA https://www.hsa.gov.sg/docs/default-source/hprg-mdb/gudiance-documents-for-medical-devices/regulatory-guidelines-for-software-medical-devices---a-life-cycle-approach.pdf (2020)), based on existing literature and well-established clinical guidelines; comparison with similarly established software medical devices in the market; and/or clinical studies for novel claims. | For apps qualifying as medical device, technical aspects on safety and performance are to follow GN-16: Guidance on Essential Principles for Safety and Performance of Medical Devices (HSA <https://www.hsa.gov.sg/docs/default-source/hprg-mdb/gn-16-r2-guidance-on-essential-principles-for-safety-and-performance-of-medical-devices(18jun-pub).pdf> (2018)) or the Essential Principles of Safety and Performance of Medical Devices and IVD Medical Devices issued by the International Medical Device Regulators Forum (<http://www.imdrf.org/docs/imdrf/final/technical/imdrf-tech-181031-grrp-essential-principles-n47.pdf> (2018)). Risk management for safe app use are to follow ISO 14971 Medical Devices: Application of Risk Management to Medical Devices (<https://www.iso.org/standard/72704.html> (2019)). | Brief cybersecurity recommendation is provided as part of the Regulatory Guidelines for Software Medical Devices: A Life Cycle Approach, including secure device design, proper customer security documentation, cyber risk management, verification and validation testing and, planning for surveillance and timely detection of emerging threats. | No existing framework or guidance, however design and manufacturing principles for medical device safety and performance include guidance that covers some aspects of usability such as the need for clear labelling and instructions, which would apply to health apps qualified as medical devices. |
| Sweden | The Swedish Medical Products Agency (SMPA) has published regulations with regards to apps considered medical products (Act (1993: 584) on medical devices. 1. Sweden (<https://www.riksdagen.se/sv/dokument-lagar/dokument/svensk-forfattningssamling/lag-1993584-om-medicintekniska-produkter_sfs-1993-584>); Law with supplementary provisions to the EU regulation on medical devices (SFS 2021: 600) (in Swedish) (https://svenskforfattningssamling.se/sites/default/files/sfs/2021-06/SFS2021-600.pdf?utm_campaign=lv_nyhetsbrev&utm_medium=email&utm_source=newsletter). To complement these, the Swedish version of the ISO standard (SIS-CEN ISO/TS 82304-2:2021 <https://www.iso.org/standard/78182.html> (2021)), which also covers wellness apps, further suggests cost of app, users should be asked on consent with regards to advertisements and use of data, and the description provided in the app store should be accurate and clear. The user should understand how the app achieves its decisions. Additional ‘product information’ is also suggested by ISO, e.g., contact info to person authorized to represent health app manufacturer. | In addition to SMPA regulations for apps considered medical products, the SIS-CEN includes: the evidence backing up the development of the app, appropriate interpretation of data, potential for harm and measures in place to control the health risks. | In addition to SMPA regulations for apps considered medical products, the SIS-CEN includes: Interoperability, and application size. | In addition to SMPA regulations for apps considered medical products, the SIS-CEN includes: compliance with data protection laws, protection against theft and viruses, signalling of breaches, authentication, data sharing, and maintenance. | In addition to SMPA regulations for apps considered medical products, the SIS-CEN includes: functionality, aesthetics, age restrictions, and availability in multiple languages. |
| USA | No current regulation regarding this. | If medical mobile applications are intended to be used ‘in the diagnosis of disease or other conditions, or in the cure, mitigation, treatment or prevention of disease’, they are regulated by the FDA as medical devices. Otherwise health content is not regulated. See also these references^27,28^ for details. | App stores have some requirements | Delegating to app stores, e.g., Google Play Developer Distribution agreement states that if you make your product/app available through the Google Play store, you must: ‘Protect the privacy and legal rights of users. Make users aware that personal information will be used by your app’. | No existing framework or guidance |

**Supplementary Table 5. Relevant criteria used in commercial stores (Apple app store and Google Play).**

| Country/ Criteria | Transparency | Health content | Technical content | Security/Privacy | Usability |
| --- | --- | --- | --- | --- | --- |
| Definition of criterion | Info about app to end-user, basis for consent | Evidence basis, appropriateness of measurement and interpretation | Software performance interoperability/ bandwidth requirements/ application size | Authentication/ anonymization, Protection against theft, viruses/signalling of breaches/data sharing/maintenance | Ease of installation and use/ tailoring/multiple languages |
| Apple app store (https://www.apple.com/app-store/) | The developer’s contact information should be displayed in the app. Privacy information, app description, screenshots and previews should be constantly updated and reflect accurately the app’s core experience. | Apps are prohibited to include offensive, insensitive, and upsetting content. In case the App features User-Generated content, the app must include methods and mechanisms to filter offensive content and block abusive users. Additionally, medical apps that are risky for the physical health of users will be rejected, as well as apps that spread false or inaccurate medical data. Developers are required to submit any regulatory certification and clearance that is applicable to their app in order during the revision process. | Before the revision process, developers are encouraged to make sure the apps do not have software performance issues. Additionally, they are required to provide a demo account and enable backend services during the revision period. | All apps are required to follow applicable data privacy laws. If an app is to be designed for children, the app is required to comply with applicable privacy laws around the world. Apps should not disclose to third parties’ data gathered in the health, fitness, and medical research context. Apps conducting health research must obtain consent from the participants or their guardians when applicable. Moreover, apps that conduct health research must be approved by an independent ethics review board and submit the approval during the revision process. | It is encouraged that apps run in iPhone and iPads, as well as support the Apple TV app and SiriKit and Shortcuts when applicable. Additionally, apps shouldn’t present risks for damaging devices or suggest or require modifications to system settings unrelated to the functionality of the app. |
| Google Play (https://play.google.com/store?hl=en_US&gl=US) | The developer’s contact information should be displayed in the app. Apps should clearly disclose their functionality, an identifiable icon and a persistent device notification to the user. | Apps should include proof of accreditation from a public authority. Additionally, apps should not include misleading, inaccurate or unverifiable health related information. | Before the revision process, developers are encouraged to make sure the apps do not have software performance issues. Additionally, they are required to provide a demo account and enable backend services during the revision period. | All apps are required to follow applicable data privacy laws. If an app is to be designed for children, the app is required to comply with applicable privacy laws around the world. Apps should not disclose to third parties’ data gathered in the health, fitness, and medical research context. | Not found |
